# IL‐9 Is Essential for ILC2‐Mediated but Not Th2‐ and Th17‐Cell‐Mediated Allergic Airway Inflammation in Mice

**DOI:** 10.1002/eji.70270

**Published:** 2026-08-24

**Authors:** Nikolaos D. Sidiropoulos, Franziska Ampenberger, Christoph Schlapbach, Christoph Schneider, Manfred Kopf

**Affiliations:** ^1^ Institute of Molecular Health Sciences ETH Zürich Zürich Switzerland; ^2^ Institute of Physiology University of Zürich Zürich Switzerland; ^3^ Department of Dermatology, Inselspital Bern University Hospital, University of Bern Bern Switzerland

**Keywords:** HDM‐induced asthma, IL‐9, IL‐9R‐specific antibody, ILC2, papain

## Abstract

Interleukin‐9 (IL‐9) has primarily been associated with type 2 immunity in health and disease. While its impact on innate lymphoid cells (ILC2s) is well known, the cellular targets and mechanisms underlying type 2 immunity remain incompletely understood. In this study, we report the generation of a mouse‐specific anti‐IL‐9 receptor (IL‐9R) antibody, enabling us to accurately map IL‐9R protein expression across various immune cell types. In naïve mice, IL‐9R was detectable in ILC2s, marginal zone B cells, and B1b cells. In allergic lung inflammation, a subset of CD4^+^ T cells faintly upregulated IL‐9R, whereas DCs, macrophages, eosinophils, and neutrophils remained IL‐9R‐negative. Functionally, IL‐9R was found to significantly contribute to ILC2 expansion, their effector cytokine production, and eosinophilia in the papain asthma model. However, in the context of acute and chronic HDM exposure, IL‐9R was dispensable for both allergic lung inflammation and CD4‐dependent memory‐type 2 responses. Using mixed bone marrow chimeras in which IL‐9R is selectively absent in T cells, we confirmed that IL‐9R signaling in T cells does not critically impact chronic Th2 and Th17 responses. Lastly, using mice selectively lacking ILC2s, we demonstrate that this population is the culprit of pathological type 2 immunity in the lung in both papain‐ and acute HDM‐induced asthma models. Together, our results establish that IL‐9 is a critical regulator of ILC2‐dependent type 2 immunity, while its effects on T cell‐dependent responses are minimal. These findings provide a foundation for future studies exploring the therapeutic modulation of IL‐9 signaling in allergy and other inflammatory diseases.

## Graphical abstract

Autocrine IL‐9 production by ILC2s promotes their expansion and effector responses, including IL‐5‐driven eosinophilia, in response to airway allergens. In contrast, Th2 cell‐intrinsic IL‐9R signaling is dispensable for type 2 lung inflammation. Lung monocytes, macrophages, and DCs do not respond to IL‐9 in asthma models due to the absence of IL‐9R expression.

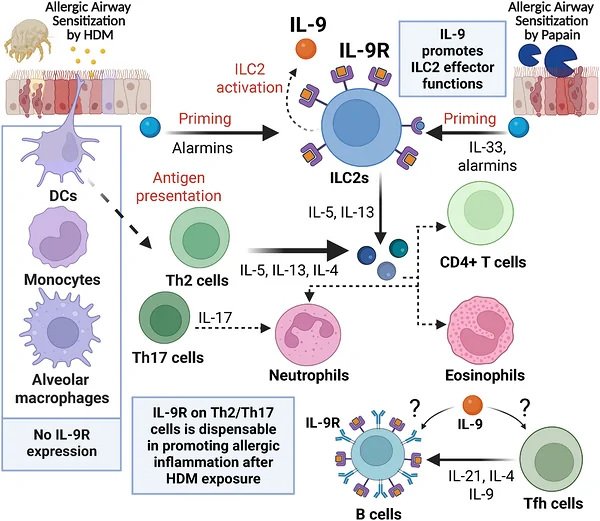

## 1 Introduction

Interleukin‐9 (IL‐9) is a pleiotropic cytokine with diverse roles in immunity and disease [1]. However, it primarily regulates type 2 immune responses, especially in nematode defense and allergic airway inflammation. Removing or neutralizing IL‐9 delays worm clearance in models of *Nippostrongylus brasiliensis* and *Trichuris muris* infections [2, 3, 4]. On the other hand, IL‐9 is involved in the pathology of asthma [5, 6, 7, 8] and atopic dermatitis [9]. Transgenic overexpression of IL‐9 causes eosinophilic infiltration in the lungs, excess mucus production, goblet cell hyperplasia, and airway remodeling [10, 11], while blocking or deleting IL‐9 reduces airway inflammation [12, 13, 14, 15]. However, the mechanisms by which IL‐9 mediates these effects are incompletely understood.

Notable IL‐9 producers in type 2 inflammatory responses include ILC2s, Th2 cells, and Th9 cells, a subset of Th2‐like cells that do not produce IL‐4. Additionally, mast cells, eosinophils, neutrophils, and NKT cells have been reported to produce IL‐9 in asthma patients and asthma mouse models [16, 17].

Although IL‐9 was initially identified as a T cell growth factor in vitro [18], its role in modulating T cell responses remains incompletely understood. Currently, IL‐9 is thought to act in an autocrine and paracrine manner on multiple cell types [19], including T cells, ILC2s, mast cells, and B cells. In ILC2s, IL‐9 promotes their expansion, cytokine production, and survival [4, 20, 21]. ILC2s coordinate type 2 immunity by releasing IL‐5, IL‐9, and IL‐13, which drive eosinophilia, mastocytosis, and goblet cell hyperplasia [22, 23, 24]. Besides its role in type 2 responses, IL‐9 is involved in CD8^+^ T cell–mediated tumor control [25, 26], Th17‐mediated EAE [27, 28], and experimental colitis [29]. Recently, IL‐9 produced by follicular helper T (Tfh) cells was shown to be necessary for the development of memory B cells in the germinal center and for recall antibody responses [10, 30, 31]. Therefore, IL‐9 influences both innate and adaptive immunity by affecting the survival, activation, and function of various immune cell types.

Despite major advances in understanding the physiological and pathological roles of IL‐9R signaling, the lack of appropriate tools has hindered detailed study of IL‐9R signaling and the elucidation of the exact connection between IL‐9R activity and phenotypic outcomes in mouse models. To address this, we developed a monoclonal antibody against the mouse IL‐9 receptor, enabling precise analysis of its expression across immune cell populations. Our results showed that IL‐9R expression is mainly limited to ILC2s, B cells, and a subset of activated T cells, but not on lung macrophages. Using papain and HDM challenge models, we found that IL‐9 signaling is a key driver of ILC2‐driven type 2 immune responses, while it is dispensable for CD4^+^ T cell‐driven acute and memory type 2 responses.

## 2 Results

### 2.1 IL‐9R Is Expressed on the Surface of ILC2s and B Cells, but Not on DCs, Monocytes, and Macrophages in the Spleen and Lung of Naïve Mice

**FIGURE 1:**
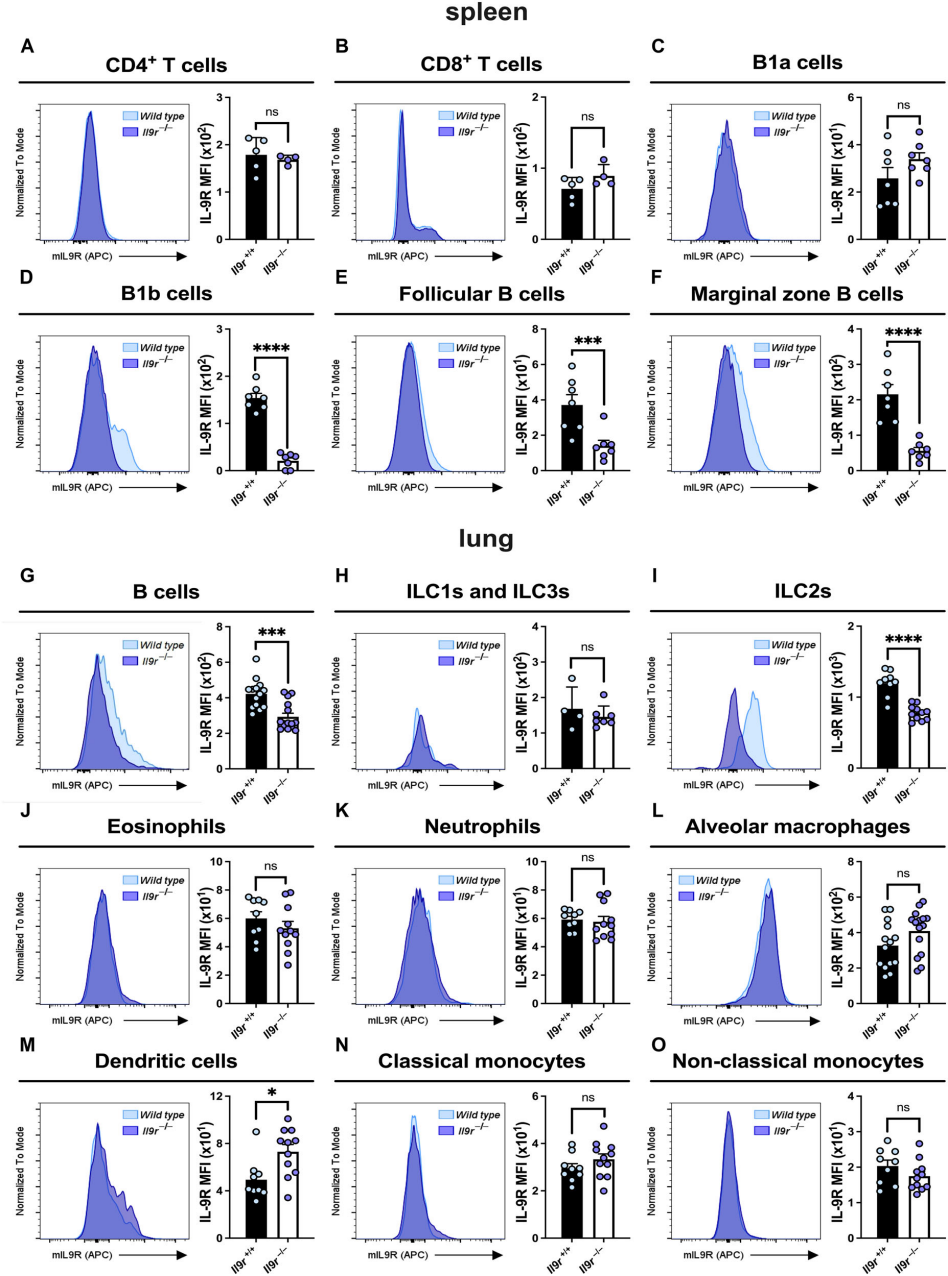
(A–O) Geometric mean fluorescence intensity (gMFI) of IL‐9R on the indicated immune cell types, stained with an anti‐mouse IL‐9R IgG1 mAb (clone 4F9C6.19): (A–F) splenocytes and (G–O) lung cells from naïve C57BL/6 wild‐type and negative control *Il9r* ^–/–^ mice. (A–O) Histograms from representative mice (left) and columns showing the group averages with symbols indicating individual mice (right). Data are shown either as mean ± SD when representative, or as mean ± SEM when pooled. Statistical analyses were performed using an unpaired *t*‐test. Data are either representative of two independent experiments (A, B, H) or pooled from two independent experiments (C–F, G, I–O).

To identify immune cells expressing surface IL‐9R, we generated a monoclonal anti‐mouse IL‐9R antibody for flow cytometry. *Il9r* ^–/–^ mice served as negative controls throughout our experiments. Splenocytes from naïve C57BL/6 mice showed high IL‐9R levels on B2 and B1b cells, whereas it was undetectable on CD4^+^ T cells, CD8^+^ T cells, and B1a cells (Figure 1A–D; Figures S1A,B and S2B). Among B2 cells, marginal zone (MZ) B cells exhibited higher levels than follicular (Fo) B cells (Figure 1E,F). Analysis of B1 cells in the peritoneal cavity confirmed IL‐9R on B1b but not B1a cells (Figure S2C). These findings mirror the pattern of *Il9r* mRNA levels identified by bulk mRNA sequencing of splenocytes, as shown in the ImmGen atlas [32] (Figure S2A). To examine IL‐9R expression in tissue immune cells, we selected the lung. Similar to the spleen, IL‐9R was expressed on CD19^+^ B cells (Figure 1G). Moreover, IL‐9R was stained on ILC2s, but it was undetectable on other innate and adaptive lymphocytes in naïve lungs (Figure 1H,I; Figures S1A and S2D). Notably, IL‐9R expression on lung ILC2s and splenic MZB cells from heterozygous *Il9r* ^+/–^ mice was about 50% lower compared to homozygous *Il9r* ^+/+^ mice, indicating a gene dosage effect (Figure S2E,F). However, IL‐9R was undetectable in the lung myeloid compartment, including alveolar macrophages (AMs), eosinophils, neutrophils, monocytes, and cDCs (Figure 1J–O; Figure S1C). In contrast to our findings, using a commercially available anti‐human IL‐9R antibody (clone AH9R7, BioLegend), Fu et al. reported that AMs, DCs, and basophils are the main IL‐9R^+^ cells in naïve mouse lungs. However, when we tested the same antibody, we could not detect IL‐9R on mouse ILC2s, B cells, or AMs, although IL‐9R was present on the human T cell line MOLT‐3, which served as a positive control (Figure S3A–C). Thus, we found no evidence that this anti‐human IL‐9R antibody cross‐reacts with mouse IL‐9R. In summary, IL‐9R is highly expressed on lung ILC2s and on a subset of pulmonary, splenic, and peritoneal B cells.

### 2.2 IL‐9 Contributes to Acute Pathological Type 2 Immunity in a Model of Papain‐Induced Asthma

**FIGURE 2:**
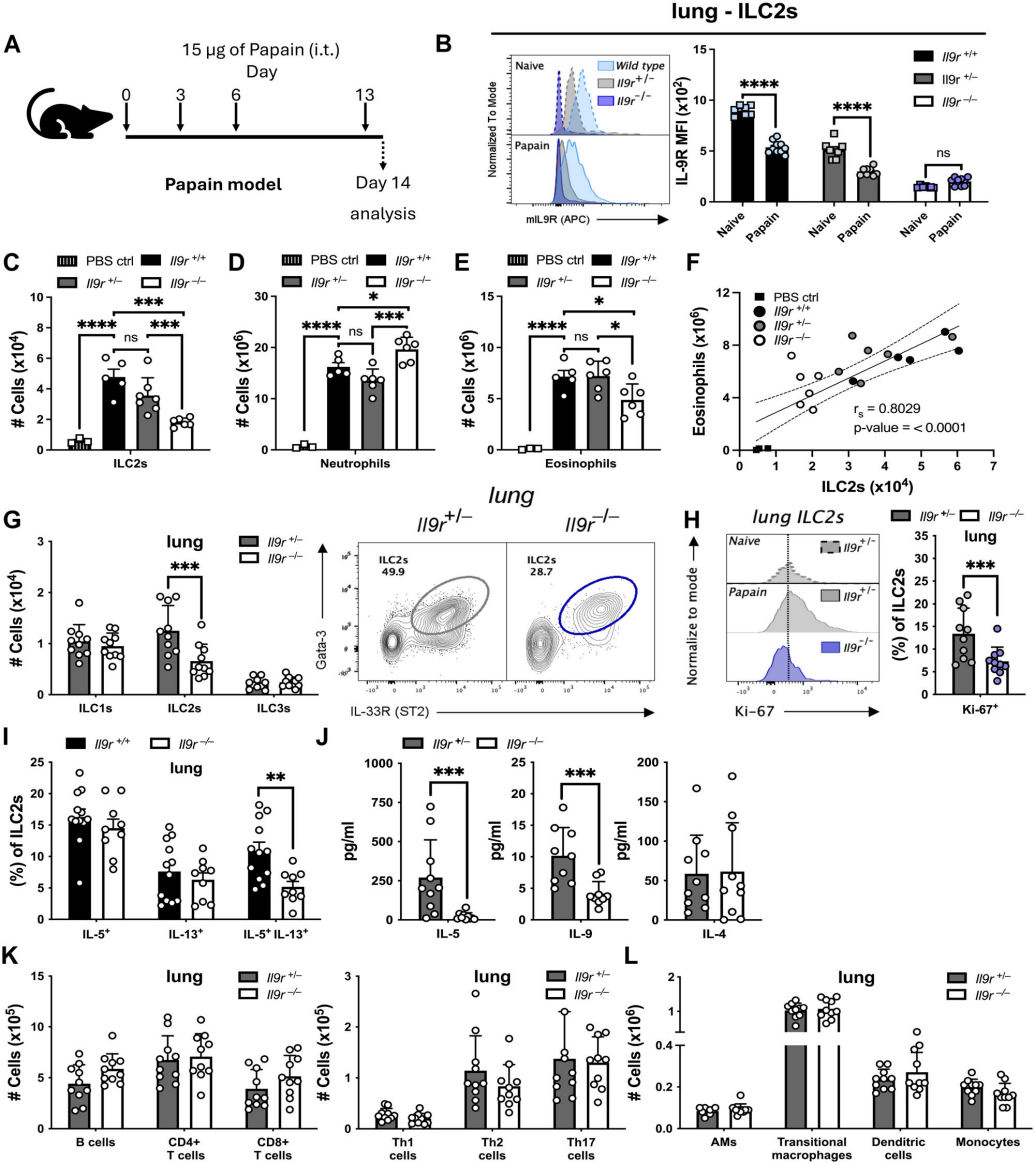
(A) Schematic overview of the papain model. Groups of mice with the indicated genotypes were treated with 15 µg papain extract or PBS (naïve) on the indicated days and analyzed on day 14. (B) Geometric mean fluorescence intensity (gMFI) of IL‐9R on lung ILC2s in naïve and papain‐treated mice. Histograms from representative mice (left) and columns showing the group averages with symbols indicating individual mice (right). (C–E) Total cell numbers of (C) ILC2s, (D) neutrophils, and (E) eosinophils in the lung. (F) Linear regression showing correlation between ILC2s and eosinophils of naïve and papain‐treated mice. (G) Total cell numbers of indicated innate lymphocyte subsets in the lung (left) and representative flow cytometry dot plot of lung ILC2s (right). (H) Representative histograms (left) and frequency of Ki‐67^+^ among ILC2s (right). (I) Frequency of the indicated cytokine‐producing ILC2s after ex vivo restimulation (PIM: PMA, Ionomycin, and Monensin) of lung cells. (J) Concentrations (pg/mL) of indicated cytokines in the BAL. (K) Total cell numbers of adaptive lymphocytes and indicated CD4^+^ T‐cell subsets (Th1:CD4^+^Tbet^+^, Th2:CD4^+^Gata3^+^, Th17: CD4^+^RORγt^+^) in the lung. (L) Total cell numbers of indicated myeloid cell populations in the lung. Data are shown either as mean ± SD when representative or as mean ± SEM when pooled. Statistical analyses were performed using an unpaired *t*‐test. Data are representative of either two independent experiments (B–F), three independent experiments (G–H, J–L), or pooled from two independent experiments (I).

Because IL‐9R was most highly expressed on the surface of ILC2s compared with other characterized immune cell types in the lung, we next examined an asthma model in which ILC2s play a key role [21, 33, 34, 35]. *Il9r* ^+/+^, *Il9r* ^+/–^, and *Il9r* ^–/–^ mice were sensitized and challenged with papain and analyzed one day after the last challenge [21] (Figure 2A). Notably, IL‐9R expression on ILC2s was downregulated upon papain exposure (Figure 2B). The absence of IL‐9R resulted in a reduced number of ILC2s, which correlated with impaired eosinophilia and type 2 effector cytokine levels in the BAL (Figure S5A), consistent with a previous report describing IL‐9 blockade [21], while the number of neutrophils was moderately elevated (Figure 2C–F). Although we again found a gene dosage‐related IL‐9R expression on ILC2s (Figure 2B), the numbers of eosinophils and ILC2s were comparable in the lungs of *Il9r* ^+/+^ and *Il9r* ^+/–^ mice (Figure 2C,E), indicating that the monoallelic IL‐9R expression did not result in a functional difference. Therefore, we continued to compare heterozygous (*Il9r* ^+/–^) and knockout (*Il9r* ^–/–^) mice from here on. Additional experiments confirmed the reduction of ILC2s (Figure 2G) and eosinophils (Figure S5B). ILC2s in IL‐9R‐deficient mice showed impaired expansion, associated with reduced Ki‐67 expression, while ILC1s and ILC3s remained unaffected (Figure 2G,H; Figure S4A). In addition, the frequencies of IL‐5^+^IL‐13^+^ ILC2 double producers, as well as levels of IL‐5 and IL‐9, were reduced (Figure 2I,J; Figure S5C). Notably, IL‐4 levels in the BAL were comparable, indicating that this type 2 cytokine is mainly produced by Th2 cells, which are unaffected in *Il9r* ^–/–^ mice (Figure 2J,K; Figures S4A and S5D), although we cannot rule out IL‐4 production by basophils and NKT cells. Importantly, the numbers of monocytes, DCs, transitional macrophages, and alveolar macrophages in the lung were comparable between *Il9r*‐deficient and control mice, consistent with the absence of IL‐9R expression on these myeloid cell types (Figure 2L; Figure S4B). Taken together, our results demonstrate that IL‐9R promotes the expansion and effector responses of ILC2s, such as eosinophilia, in a papain model, similar to observations in the lungs after *N. brasiliensis* infection [4].

### 2.3 IL‐9 Promotes ILC2‐Mediated, but Not CD4 T Cell‐Mediated, Allergic Inflammation Following Chronic HDM Exposure

**FIGURE 3:**
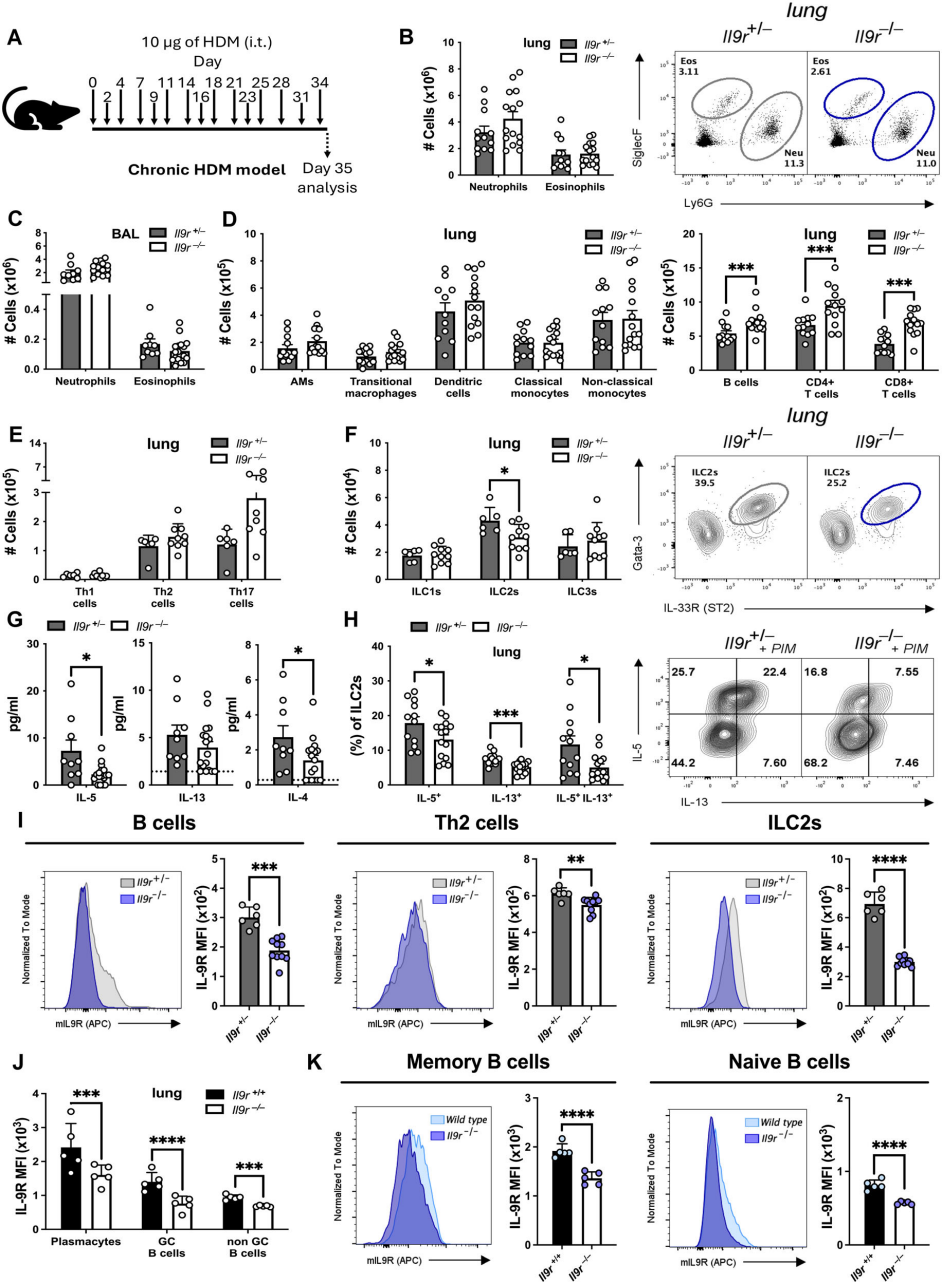
(A) Schematic overview of the chronic HDM model, where *Il9r* ^+/–^ (grey) and *Il9r* ^–/–^ (white) littermates were treated with 10 µg HDM extract three times per week and analyzed after 5 weeks. (B–C) Total cell numbers of indicated myeloid cell populations in (B) the lung (left panel) and representative flow cytometry dot plots (right panel), and in (C) the BAL. (D) Total cell numbers of myeloid cells and adaptive lymphocytes in the lung. (E) Total cell numbers of indicated CD4^+^ T‐cell subsets (Th1:CD4^+^Tbet^+^, Th2: CD4^+^Gata3^+^, Th17:CD4^+^RORγt^+^) in the lung. (F) Total cell numbers of indicated innate lymphocyte subsets in the lung (left) and representative flow cytometry dot plot of lung ILC2s (right). (G) Concentrations (pg/mL) of indicated cytokines in the BAL. (H) Frequency of cytokine‐producing ILC2s after short ex vivo restimulation (PIM: PMA, Ionomycin and Monensin) of lung cells and a representative flow cytometry dot plot of pregated lung ILC2s showing IL‐5 and IL‐13 expression. (I–K) Geometric mean fluorescence intensity (gMFI) of IL‐9R on indicated immune cell types using an anti‐mouse IL‐9R IgG1 mAb (clone 4F9C6.19) to stain lung (I) B‐cell subsets, Th2 cells and ILC2s and (J, K) B‐cell subpopulations from chronic HDM‐treated *Il9r* ^+/–^ and negative control *Il9r* ^–/–^ mice. Histograms from representative mice (left) and columns showing the group averages with symbols indicating individual mice (right). Lymphocytes were gated according to Figure S4A, myeloid cells according to Figure S4B, and B cells according to S6A. Data are shown either as mean ± SD when representative or as mean ± SEM when pooled. Statistical analyses were performed using an unpaired *t*‐test. Data are either representative of two or three independent experiments (E, F, I–K) or pooled from two independent experiments (B–D, G, H).

IL‐9 signaling has been suggested to play a critical role in models of acute and chronic airway exposure to house dust mite (HDM) [12, 13, 14, 36]. To better characterize IL‐9‐responsive cells in these models, we first treated *Il9r* ^–/–^ and littermate *Il9r^+/–^* control mice with a low dose (10 µg) HDM over five weeks (Figure 3A). In asthmatic mice, the granulocyte infiltrate in the BAL and lung consisted of both eosinophils and neutrophils, with neutrophils being more abundant, consistent with previous reports in the chronic HDM model [37, 38]. However, their numbers in the BAL and lung were comparable between *Il9r* ^–/–^ and littermate control mice (Figure 3B,C). The number of other myeloid cells, including alveolar macrophages, monocyte‐derived transitional macrophages, and DCs, also remained unaffected in the absence of IL‐9R (Figure 3D). Among lymphocytes, the numbers of B cells, CD4^+^ and CD8^+^ T cells were slightly increased, whereas ILC2s were markedly reduced in the lungs of *Il9r* ^–/–^ mice (Figure 3D–F). The IL‐4 and IL‐5 protein levels in the BAL fluid were decreased (Figure 3G). Additionally, ex vivo restimulation of lung cells revealed significantly reduced frequencies of type 2 cytokine‐producing ILC2s in IL‐9R‐deficient mice (Figure 3H). Similar to naïve lungs, B cells and ILC2s clearly expressed surface IL‐9R, while it was poorly expressed on CD4^+^Gata3^+^ Th2 cells in the lungs of HDM‐treated mice (Figure 3I). Among B cells, IL‐9R surface expression was observed across all subpopulations, including naïve, germinal center, and memory B cells, as well as plasma cells (PCs) in the lungs of asthmatic mice (Figure 3J,K; Figure S6A). Surprisingly, levels of HDM‐specific IgG1 and total IgE tended to be increased rather than decreased in the BAL of *Il9r* ^–/–^ mice (Figure S6D). IL‐9R was undetectable on eosinophils, neutrophils, monocytes, monocyte‐derived macrophages, AMs, DCs, CD8^+^ T cells, CD4^+^Tbet^+^ Th1 cells, and CD4^+^RORγt^+^ Th17 cells (Figure S6B,C).

To assess whether findings from the chronic model also applied to acute allergic lung inflammation, we analyzed mice in an acute HDM model with shorter allergen challenge (Figure S7A). Compared to the chronic model, acute HDM exposure led to more robust eosinophilia (Figure S7B). Similar to the chronic model, *Il9r* ^–/–^ mice showed reduced ILC2 numbers with fewer Ki‐67^+^ ILC2s (Figure S7D), while the number of other immune cells, including eosinophils, neutrophils, B cells, and T cells, was comparable in *Il9r^–/–^* and *Il9r^+/+^* mice (Figure S7C). As in the chronic model, we found the highest IL‐9R expression on ILC2s and B cells, with faint expression on Th2 and Th17 cells (Figure S7E).

### 2.4 T Cell‐Intrinsic IL‐9R Signaling Is Dispensable for Chronic HDM‐Induced Type 2 Lung Inflammation

**FIGURE 4:**
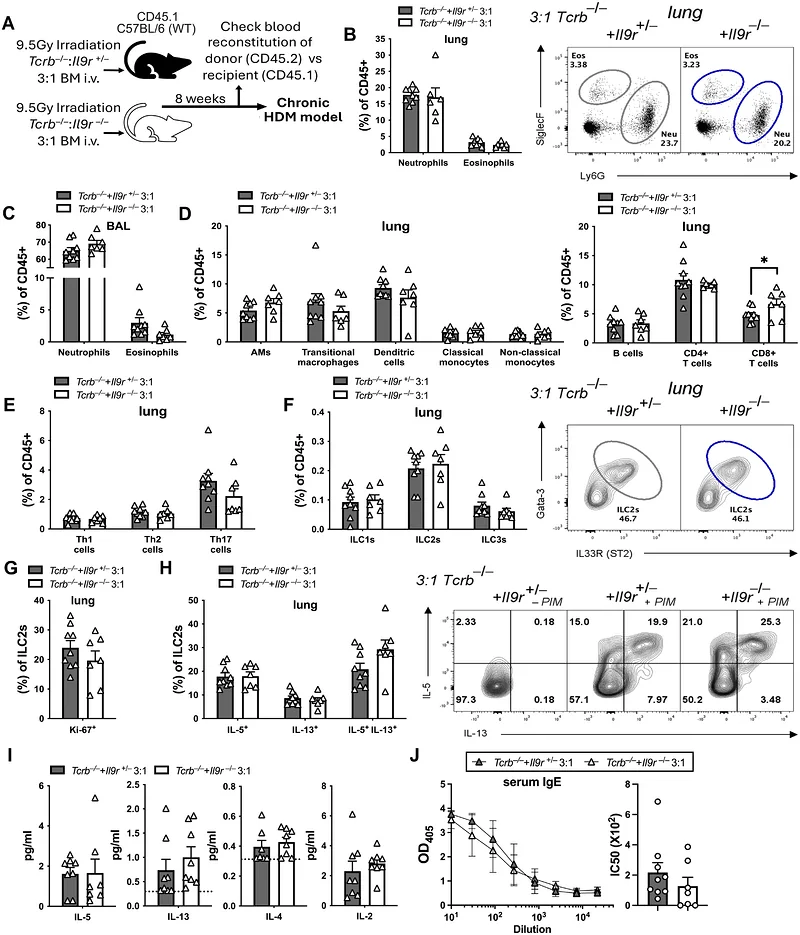
(A) Schematic overview of the experiment where lethally irradiated mice were reconstituted with bone marrow (BM) from *T* *crb* ^–/–^ mice mixed in a 3:1 ratio with BM from either *Il9r* ^+/–^ (grey) or *Il9r* ^–/–^ (white) mice to generate controls and mice that lack *Il9r* specifically on T cells, respectively. Mice were challenged with 10 µg of HDM three times per week for five weeks and analyzed on day 35. (B–C) Frequency among CD45^+^ cells of indicated myeloid cell populations in (B) the lung (left panel) and representative flow cytometry dot plots (right panel), and in (C) the BAL. (D) Frequency among CD45^+^ cells of myeloid cells and adaptive lymphocytes in the lung. (E) Frequency among CD45^+^ cells of indicated CD4^+^ T‐cell subsets (Th1:CD4^+^Tbet^+^, Th2: CD4^+^Gata3^+^, Th17:CD4^+^RORγt^+^) in the lung. (F) Frequency among CD45^+^ cells of indicated innate lymphocyte subsets in the lung (left) and representative flow cytometry dot plot of lung ILC2s (right). (G) Percentage of Ki‐67^+^ among ILC2s. (H) Frequency of cytokine‐producing ILC2s after short ex vivo restimulation (PIM: PMA, Ionomycin and Monensin) of lung cells and a representative flow cytometry dot plot of pre‐gated lung ILC2s showing IL‐5 and IL‐13 expression. (I) Concentrations (pg/mL) of indicated cytokines in the BAL. (J) Total serum IgE levels. Data are shown as mean ± SEM. IC_50_ values were calculated for each mouse by nonlinear regression (four‐parameter logistic fit) of ELISA dilution curves. Statistical analyses for (B–I) were performed using an unpaired *t*‐test. Data are pooled from two independent experiments.

To determine whether CD4^+^ T cell‐intrinsic IL‐9R signaling contributes to allergic lung inflammation, we generated mice that lack IL‐9R specifically on T cells. To this end, lethally irradiated wild‐type C57BL/6 (WT) recipients were treated with a 1:3 mixture of bone marrow from either *Il9r^–/–^* and *Tcrb^–/–^* mice (T*^Il9r^* ^–/–^), or *Il9r^+/–^* and *Tcrb^–/–^* mice (T*^Il9r^* ^+/–^) as a control. After reconstitution of the immune system, the chimeras were chronically exposed to HDM, as described previously (Figure 4A). Consistent with our previous experiments using global *Il9r* ^–/–^ mice, the absence of IL‐9R in T cells only did not lead to major alterations in inflammatory cell infiltration, including unchanged numbers of ILC2s (Figure 4B–F). The frequencies of Ki‐67^+^ and cytokine‐producing ILC2s and Th2 cells were also comparable (Figure 4G,H; Figure S8C,D). Additionally, type 2 cytokine levels in BAL fluid and serum IgE levels did not differ significantly between the two groups (Figure 4I,J). Together, these data demonstrate that T cell‐intrinsic IL‐9R signaling does not affect type 2 inflammation in the lung.

### 2.5 ILC2s Are Key for Lung Pathological Type 2 Immunity Upon Exposure to both Papain and HDM

**FIGURE 5:**
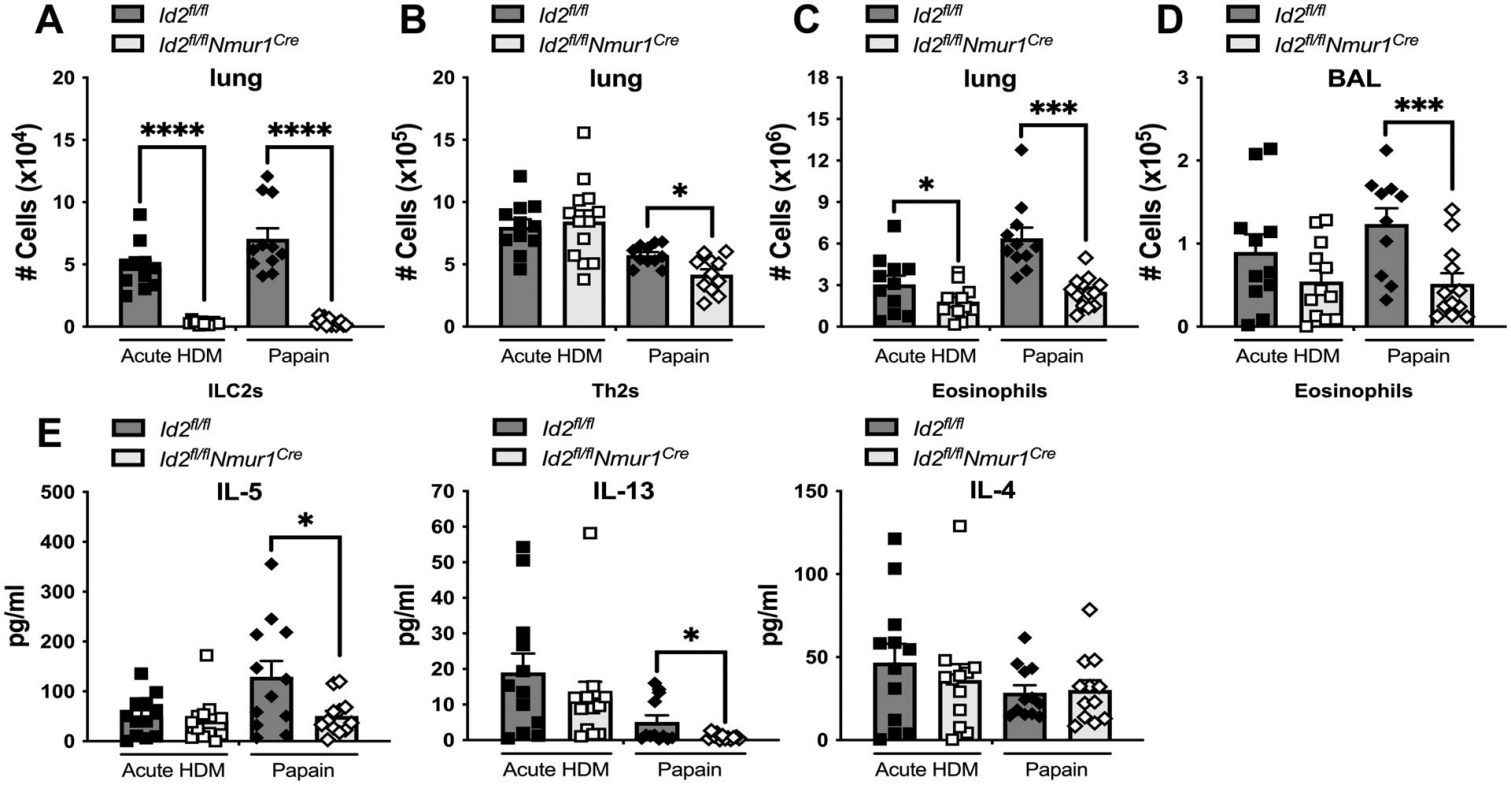
(A–D) Quantification of ILC2s in the lung (A), Th2 cells in the lung (B), eosinophils in the lung (C), and BAL (D) of *Id2* ^fl/fl^ (grey) and *Id2* ^fl/fl^ *;Nmur1* ^Cre/+^ (white) mice after acute HDM and papain treatment. (E) Cytokine concentrations (pg/mL) of IL‐5, IL‐13, and IL‐4 in the BAL. Data are shown as mean ± SEM. Statistical analyses were performed using an unpaired *t*‐test. Data are pooled from two independent experiments.

To compare the role of ILC2s in promoting type 2 inflammation following acute HDM and papain treatment, we employed *Id2* ^fl/fl^;*Nmur1* ^Cre/+^ mice, which lack functional ILC2s due to impaired development [35, 39]. In the *Id2* ^fl/fl^ control group, we observed robust ILC2 expansion in response to papain, while Th2 cell differentiation predominated in the acute HDM model, highlighting the distinct cellular drivers of type 2 inflammation in each model (Figure 5A,B). In the papain model, loss of ILC2s led to a substantial reduction in eosinophilia, Th2 differentiation, and cytokine production. Eosinophilia was also impaired in the acute HDM model, though to a lesser extent, indicating that ILC2s contribute partially to this T cell‐dependent response (Figure 5B–E). Overall, these results confirm the central role of ILC2s in papain‐driven type 2 immunity and reveal a modest contribution in HDM, which may underlie the phenotype in *Il9r*‐deficient mice.

## 3 Discussion

Interleukin‐9 (IL‐9) is a crucial cytokine in type 2 immunity. While it is beneficial for efficient clearance of gastrointestinal nematodes [2, 3, 4], it has been suggested to act detrimentally by promoting type 2 allergic lung inflammation [5, 6, 7, 8]. However, previous studies have not rigorously assessed IL‐9R cell surface expression or its role in modulating the function of CD4^+^ T cells, ILC2s, and myeloid cells in allergic lung disease. Using a novel anti‐IL‐9R antibody, our study provides a detailed profile of IL‐9R expression across immune cell types and clarifies their roles in various asthma models.

Our results demonstrate that IL‐9R is predominantly expressed by B cells and ILC2s in the lungs of naïve and HDM‐allergic mice. While IL‐9R was undetectable on CD4+ T cells in naïve mice, it was slightly upregulated on Th2 and Th17 cells upon HDM exposure. In contrast to previous reports suggesting that lung macrophages responding to IL‐9 play key roles in type 2 allergic lung inflammation and in lung tumor models [40, 41], we found no evidence that alveolar macrophages, DCs, monocytes, or monocyte‐derived cells express IL‐9R in HDM‐allergic lungs. Additionally, neutrophils and eosinophils were IL‐9R‐negative. Notably, the use of species‐specific reagents and knockout validation allowed us to overcome the limitations of prior studies that relied on anti‐human IL‐9R antibodies [40], which we showed did not accurately detect IL‐9R expression on mouse cells.

Several studies using different animal models have concluded that IL‐9 promotes allergic lung inflammation (reviewed in [6, 42]). Our results demonstrate that, following papain exposure, the IL‐9 pathway enhances acute lung eosinophilia, but not neutrophilia, by promoting ILC2 effector responses, confirming and extending a previous report [21]. Our data indicate that IL‐9 drives ILC2 proliferation, whereas another study concluded that it supports Bcl3‐mediated ILC2 survival [4]. The differences may be explained by the distinction between ex vivo and in vivo EdU labeling of ILC2s.

While optimal accumulation of ILC2 effectors in the lung also depends on IL‐9 following acute and chronic HDM exposure, in this case, IL‐9 is dispensable for the recruitment of eosinophils and neutrophils, likely due to IL‐9‐independent development of Th2 and Th17 cells. However, in ILC2‐deficient (*Id2* ^fl/fl^ *;Nmur1* ^Cre/+^) mice, eosinophilia and type 2 cytokine production were impaired in the HDM model, indicating that ILC2s partially contribute to allergic lung inflammation [35]. HDM can directly activate ILC2s via TLR4 [43]. By generating chimeric mice lacking IL‐9R specifically in T cells, we confirmed that CD4^+^ T cell‐intrinsic IL‐9R signaling is not required for adaptive Th2 responses and associated lung inflammation induced by chronic HDM exposure.

A recent report consistently showed that IL‐21, but not IL‐9, is responsible for Th2 responses and allergic lung inflammation upon chronic HDM exposure [44]. Likewise, neutralization of IL‐9 during the last week of chronic exposure to the allergen *Aspergillus fumigatus* (*A.f*.) did not affect the inflammatory infiltrate, whereas IL‐9 produced by memory CD4^+^ T cells was required for allergic inflammation in a recall response several weeks after chronic *A.f*. exposure [36]. It remains to be studied whether T‐cell‐intrinsic IL‐9R signaling is necessary for the development of memory CD4^+^ T cells in the *A.f*. model.

Overall, these results suggest that the role of IL‐9 in allergic lung inflammation depends on the model and context. The use of different allergens, HDM batch and dosage, and genetic backgrounds of the IL‐9/IL‐9R KO mice (C57BL/6 versus BALB/c) in the studies may explain the variability in results and conclusions [12, 13, 14, 15].

In addition to ILC2s, different B cell subsets in naïve and HDM‐treated mice showed the highest IL‐9R expression. Innate‐like B‐1 cells, which predominantly reside in serosal cavities, can be separated into two subtypes, B1a and B1b, based on CD5 expression (i.e., CD5^+^ B1a and CD5^–^ B1b). The two subtypes also differ in functional features and origin [45, 46, 47, 48]. Our data revealed that these subtypes can also be distinguished by IL‐9R expression, which is high in B1b cells and undetectable in B1a cells in the peritoneum and the spleen. B1b cells also express IL‐5R, IL‐4R, and IL‐6R (ImmGen), and steady‐state IL‐5 production by ILC2s has recently been shown to be essential for the development and function of B1a and B1b cells [49]. However, transgenic overexpression of IL‐9 results in a striking expansion of B1b cells and increased baseline and antigen‐specific antibody levels across all isotypes [50], indicating that IL‐9 is particularly important for B1b responses. Amongst naïve splenic B2 cells, marginal zone (MZ) B cells express higher levels compared to follicular B cells. Similar to B1 cells, MZB cells are innate‐like B cells that mediate rapid T‐cell‐independent IgM and IgG responses to bacterial infections [51, 52]. Consistent with recent publications highlighting the critical role of IL‐9 derived from follicular helper T (Tfh) cells in recall antibody responses following immunization with NP‐chicken‐γ‐globulin (NP‐CGG), NP‐KLH, and sheep red blood cells (SRBC) by memory B cells [10, 31, 53], we found that IL‐9R is highly expressed on both memory and germinal center B cells. However, surprisingly, in contrast to these studies, IL‐9R was dispensable for IgE and IgG1 antibody responses following chronic airway HDM exposure. Also, IgE responses following HDM treatment were unaffected in mice lacking IL‐9R specifically in T cells, implying that Tfh cell‐intrinsic IL‐9R signaling is not essential for their helper activity in the chronic HDM model.

### 3.1 Data Limitations and Perspectives

Clearly, further studies using different antigen types and immunization routes, along with conditional knockouts lacking IL‐9R exclusively in B cells and Tfh cells, are warranted to clarify the role of IL‐9 in B‐cell responses. Taken together, our data show that IL‐9 contributes to ILC2‐mediated allergic airway responses but is not essential for acute or chronic Th2‐ and Th17‐driven eosinophilia and neutrophilia, underscoring a context‐dependent role. The absence of IL‐9R on lung monocytes, macrophages, DCs, and granulocytes indicates that these cell types do not control lung inflammation by responding to IL‐9. Conditional gene targeting of IL‐9R in these cell types will help to definitively rule them out. Although IL‐9R expression on lung mast cells and a role in airway remodeling and hyperresponsiveness have been established in humans and mice [24, 54, 55], we have not analyzed them due to their scarcity in peripheral airways and an uncertain role in allergic inflammation in mouse models [56, 57]. Given the context‐dependent role of IL‐9 established in mice, translational advances will require studies including patients with different asthma endotypes and phenotypes to determine when IL‐9 blockade will be beneficial and when not. This knowledge will be crucial for designing tailored asthma therapies. Characterizing IL‐9R‐expressing cells will also be useful for dissecting their roles in models of autoimmunity, infection, and cancer [27, 58, 59, 60, 61].

## 4 Materials and Methods

### 4.1 Experimental Mice

*Tcrb^–/–^* (JAX, strain #002118), C57BL6/J (CD45.1, JAX, strain #002014), and C57BL6/J (CD45.2, JAX, strain #000664) were obtained from the Jackson Laboratory (Bar Harbor, Maine). *Il9r^–/–^* C57BL/6 mice [62] (kindly provided by J.C. Renauld) and *Id2* ^fl/fl^ *;Nmur1* ^Cre/+^ *mice* [39]. Mice were bred and maintained in individually ventilated cage units and under specific pathogen‐free conditions at the ETH Phenomics Center (EPIC) Zurich. Animals were maintained on a constant 12 h light/dark cycle, with chow and water provided ad libitum. Experiments were conducted with age‐matched animals, 6–16 weeks old, evenly distributed between males and females.

### 4.2 Mouse Treatments

Mice were anesthetized with isoflurane and repeatedly instilled intratracheally (i.t.) with 50 µL of allergen solution (i.e., 15 µg papain (Sigma‐Aldrich, #9001‐73‐4) or 10 µg house dust mite (HDM) (*Dermatophagoides pteronyssinus* extract, Citeq Biology) as illustrated in the treatment schemes. For analysis, mice were sacrificed by i.p. injection of a sodium pentobarbital overdose.

### 4.3 Generation of a Monoclonal Anti‐Mouse IL‐9R Antibody

To generate IL‐9R‐hIgG1/Fc fusion protein constructs, the extracellular domains of IL‐9R and IL‐2Rγ were amplified by PCR and cloned upstream of a human IgG1 Fc sequence. The constructs were co‐transfected into HEK 293EBNA cells, and heterodimeric fusion proteins were purified using protein A chromatography. *Il9r* ^–/–^ mice were immunized with 100 µg of the heterodimeric fusion protein in CFA and boosted on day 14 with 50 µg of the fusion protein in IFA. To avoid strong induction of anti‐hIgG1 antibodies, mice were subsequently injected with BEKO cells overexpressing native membrane‐bound IL‐9R. Production of IL‐9R‐specific antibodies was confirmed by staining BEKO and HEK293T cells transfected with a mouse IL‐9R expression vector. Next, splenocytes were fused with SP2/0 myeloma cells per standard protocols, and positive clones were identified by screening supernatants on IL‐9R‐overexpressing BEKO cells. Untransduced BEKO cells and cells from *Il9r* ^–/–^ mice served as negative controls. Selected clones were subcloned twice and expanded to collect supernatant. Antibody (clone 4F9C6.19) was purified from supernatant using protein G chromatography. For flow cytometry, an APC‐conjugated goat anti‐mouse antibody (Southern Biotech, catalog number #1070/11L) was used to stain surface IL‐9R.

### 4.4 Generation of Bone Marrow Chimeras

C57BL/6 recipient mice were lethally irradiated with 9.5 Gy (administered as two doses of 4.75 Gy with a 4 h break in between) using an RS2000 machine (Rad Source Technologies Inc., Alpharetta, USA). The next day, bone marrow cells were obtained from donor mice by flushing the femurs and tibias. Red blood cells were lysed with ammonium‐chloride‐potassium buffer (ACK, homemade) and then counted. The number of injected cells was normalized to 3 million per recipient mouse and injected in 200 µL endotoxin‐free PBS intravenously after warming up mice to 37 °C.

### 4.5 Tissue and Cell Suspension Preparation

Mice were euthanized with sodium pentobarbital (400 mg/kg). To collect bronchoalveolar lavage fluid (BALF), an 18‐G venous catheter (BD Insyte) was inserted into the trachea through a small incision. For BALF collection, the lungs were flushed three times with 0.8 mL PBS containing 2 mM EDTA (Sigma‐Aldrich), and the fluid was subsequently retrieved. BALF was centrifuged at 350 x g for 5 min, the supernatants were collected, and the cell pellets were resuspended in PBS with 2% FCS (Gibco).

Blood was collected from the vena cava and placed in a Multivette‐600 tube (Sarstedt) for serum separation. For staining of blood cells, 50 µL of blood was collected from the tail vein and mixed with Liquemin‐heparin to prevent coagulation. Red blood cells were lysed with ACK buffer, centrifuged, and the pellet was resuspended in PBS with 2% FCS for further staining.

Lungs were perfused with 10 mL of cold PBS through the right ventricle of the heart, then harvested and stored in PBS with 2% FCS on ice. After dissection, lung samples were minced using a gentleMACS dissociator (Miltenyi Biotec) and enzymatically digested in Iscove's Modified Dulbecco's Medium (Gibco) with 600 U/mL Collagenase IV (Worthington) and 200 U/mL DNase I (Worthington) for 45 min at 37°C under constant agitation at 130–150 rpm. Following a second homogenization with the gentleMACS dissociator (program m_lungs_02_01), samples were strained through 70 µm filters, erythrocytes were lysed by incubation with an ammonium‐chloride‐potassium (ACK) buffer for 5 min at room temperature, and the resulting single‐cell suspension was passed through a 40 µm strainer to obtain a cell suspension free of clumps.

To analyze the B cell populations, the lungs were removed and placed into gentleMACS tubes containing PBS with 2% FCS (Gibco). The lungs were cut into small pieces by hand with scissors. The samples were passed through a 100 µm strainer, erythrocytes were lysed by incubation in ammonium‐chloride‐potassium (ACK) buffer for 5 min at room temperature (RT), and the resulting single‐cell suspension was filtered through a 40 µm strainer to obtain a uniform cell suspension.

### 4.6 Restimulation

For cytokine staining, cells were restimulated ex vivo with 0.1 µg/mL PMA (Sigma‐Aldrich), 1 µg/mL ionomycin (Brunschwig), and 2 µg/mL monensin (Sigma‐Aldrich) in IMDM medium containing 50 µM β‐Mercaptoethanol (Gibco), 10% FCS, and 100 U/mL penicillin/100 µg/mL streptomycin (Gibco) for 4 h at 37°C.

### 4.7 Flow Cytometry

Cells were incubated with LIVE/DEAD Fixable Blue viability dye (Thermo Fisher) and FcγRIII/II‐blocking antibody (clone 2.4G2) in PBS for 20 min at 4°C. For surface staining, cells were incubated with fluorochrome‐ or biotin‐conjugated antibodies in PBS with 2% FCS for 20 min. Before intracellular staining, cells were washed and treated with the Foxp3/Transcription Factor Fixation/Permeabilization kit (Thermo Fisher). Cells were stained for CD45 (30‐F11), CD45.1 (A20), CD45.2 (104), CD49b (HMa2), CD19 (6D5), TCRβ (H57‐597), CD4 (GK1.5), CD90.2 (30‐H12), Gata3 (L50‐823), Tbet (4B10), RORγt (B2D), IL33R (DIH9), Ki67 (11F6), KLRG1 (2F1), CD38 (90), CD95 (Sa367H8), GL7, CD11b (M1/70), CD11c (N418), MHCII (M5/114.15.2), Ly6C (HK1.4), Ly6G (1A8), F4/80 (BM8), SiglecF (E50‐2440), MERTK (DS5MMER), XCR1 (ZET), CD64 (X54‐5/7.1), IFNγ (B27), IL5 (TRFK5), IL13 (W17010B), IL4 (11B11), IL10 (JES5‐16E3) with antibodies from Biolegend and CD8 (53–6.7, eBioscience). Stained samples were acquired on a BD FACSymphony A5 SE cell analyzer equipped with 5 lasers. Data were analyzed using manual gating with FlowJo (BD Biosciences). Doublets were excluded for each population individually at the end of the gating hierarchy. Gates were kept constant across all samples unless clear changes in the population density distributions required adjustment.

### 4.8 Cytokine Quantification

Cytokine concentrations in BALF were determined using the LEGENDplex mouse Th and Th2 panel version 03 (Biolegend). For every cytokine, the limit of detection is indicated by a dashed line in the figure.

### 4.9 Statistical Analysis

Each data point represents a biological replicate. Statistical analyses were performed using GraphPad Prism (GraphPad Software Inc., Version 10.1.0) as indicated in the figure legends. All data are shown either as mean values ± standard deviation (SD) when representative of different experiments, or as mean values ± standard error of the mean (SEM) when being pooled from different experiments. *p <0.05; **p <0.01; ***p <0.001; ****p <0.0001.

## Author Contributions

N.D.S. and C.S. (Zürich) performed experiments, analyzed data, and wrote the manuscript. F.A. provided technical assistance. C.S. (Bern) supplied valuable reagents. M.K. directed the study, acquired funding, and wrote the manuscript.

## Funding

This research was supported by the ETH Zürich Project SKINTEGRITY.CH to M.K. and the Swiss National Science Foundation, Eccellenza grant (194216) to C.S.

## Ethics Statement

All studies were approved by the local animal ethics committee (Cantonal Veterinary Office, ZH156/2022; ZH121/2024) and complied with the guidelines specified in the Cantonal Animal Welfare Ordinance (KTschV, Zurich) and the Swiss Animal Welfare Act (TschG).

## Conflicts of Interest

The authors declare no conflicts of interest.

## Supporting information




**Supporting File**: eji70270‐sup‐0001‐SuppMat.pdf.

## Data Availability

All data are available in the main article, in the supplementary materials, and from the corresponding authors upon reasonable request.
