# Supplementary material for: IL‐9 Is Essential for ILC2‐Mediated but Not Th2‐ and Th17‐Cell‐Mediated Allergic Airway Inflammation in Mice
Source: Eur J Immunol. 2026 Aug 24;56(8):e70270. doi: 10.1002/eji.70270 (PMC13501938; doi:10.1002/eji.70270)
Supplement: Supplementary file 1 [file EJI-56-e70270-s001.pdf]

Supplementary Materials

IL-9 is essential for ILC2-mediated but not $CD4^+$ T cell-mediated allergic airway inflammation in mice

Nikolaos D. Sidiropoulos[1,2], Franziska Ampenberger[1], Christoph Schlapbach[3], Christoph Schneider[2], Manfred Kopf[1]

[1] Institute of Molecular Health Sciences, ETH Zürich, Zürich, Switzerland

[2] Institute of Physiology, University of Zürich, Zürich, Switzerland

[3] Department of Dermatology, Inselspital, Bern University Hospital, University of Bern, Bern, Switzerland

Correspondence: Manfred Kopf, E-mail: Manfred.Kopf@ethz.ch

Sup. Figure 1

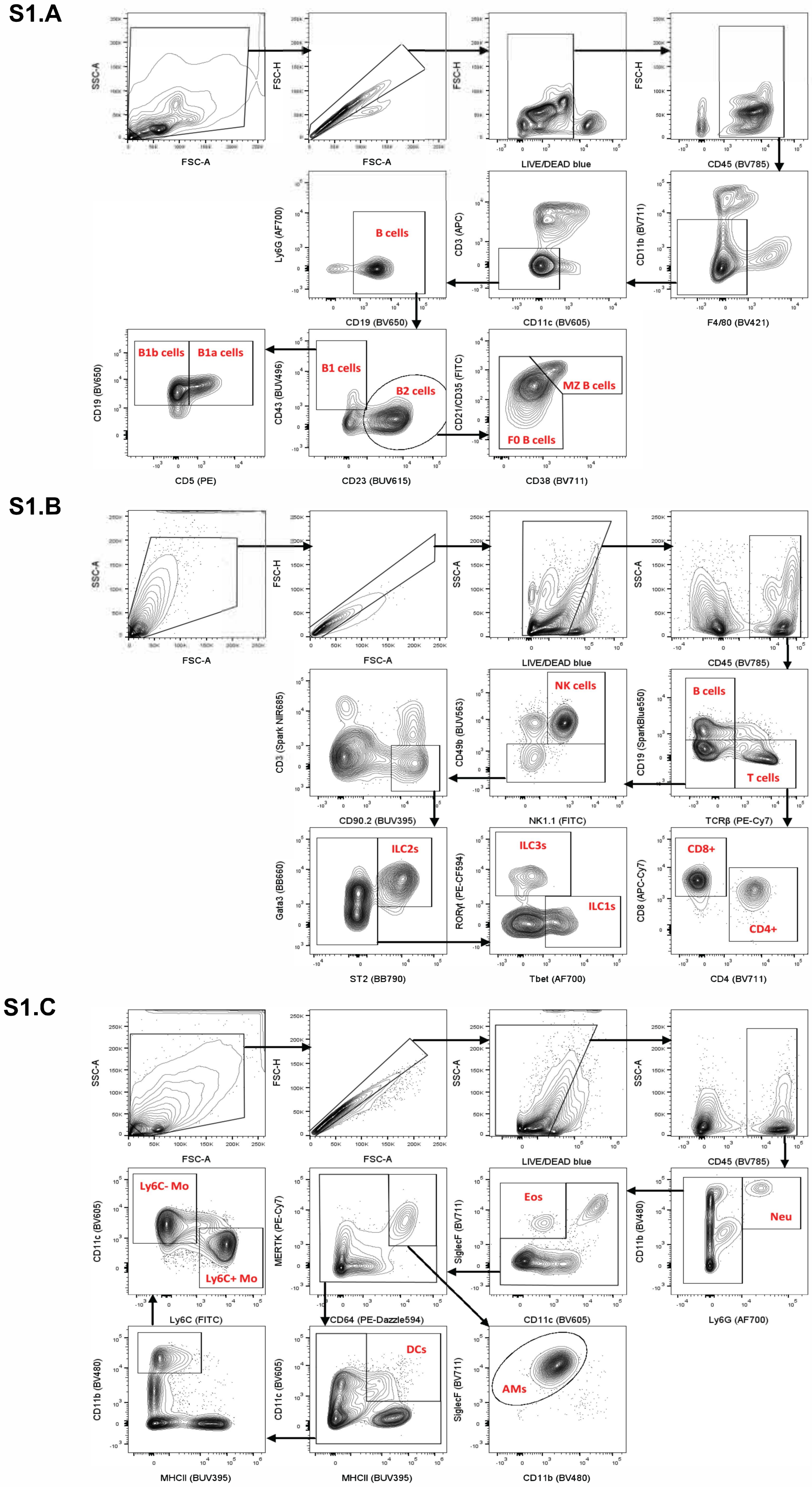

Supplementary Figure 1. A-C) Gating strategies for characterization of B cells in the spleen and peritoneum (A), lymphoid cells in the spleen and lung (B), and myeloid cells in the lung (C) of naïve C57BL/6 mice.

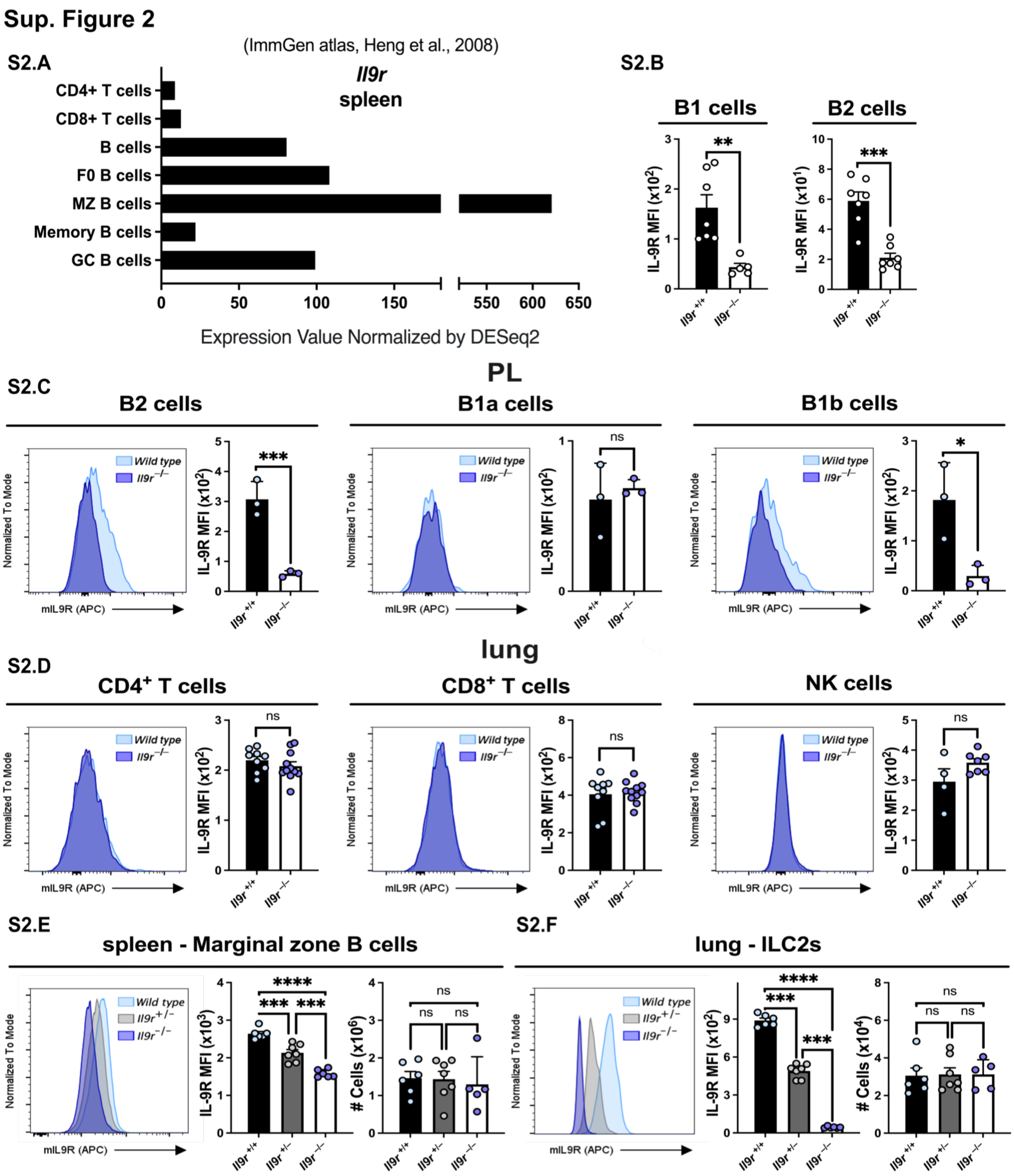

Supplementary Figure 2. A) Expression levels of *Il9r* transcripts in B-cell populations in the spleen according to ImmGen. **B-D)** Geometric mean fluorescence intensity (gMFI) of IL-9R on indicated immune cell types stained with an anti-mouse IL-9R IgG1 mAb (clone 4F9C6.19) to stain (B) splenocytes, (C) peritoneal cells, and (D) lung cells from naïve C57BL/6 wild-type and negative control *Il9r*$^{-/-}$ mice. **E-F)** Geometric mean fluorescence intensity (gMFI) of IL-9R and cell numbers of (E) splenic marginal zone B cells and (F) lung ILC2s from naïve C57BL/6 wild-type (black), *Il9r*$^{+/-}$ (grey) and negative control *Il9r*$^{-/-}$ (white) mice. (B-F) Histograms from representative mice (left) and columns showing the group averages with symbols indicating individual mice (right). Data are shown either as mean ± s.d. when representative or as mean ± s.e.m. when pooled. Statistical analyses were performed using an unpaired *t*-test. Data are either representative of two independent experiments (C, E-F) or pooled from two independent experiments (B, D).

Sup. Figure 3

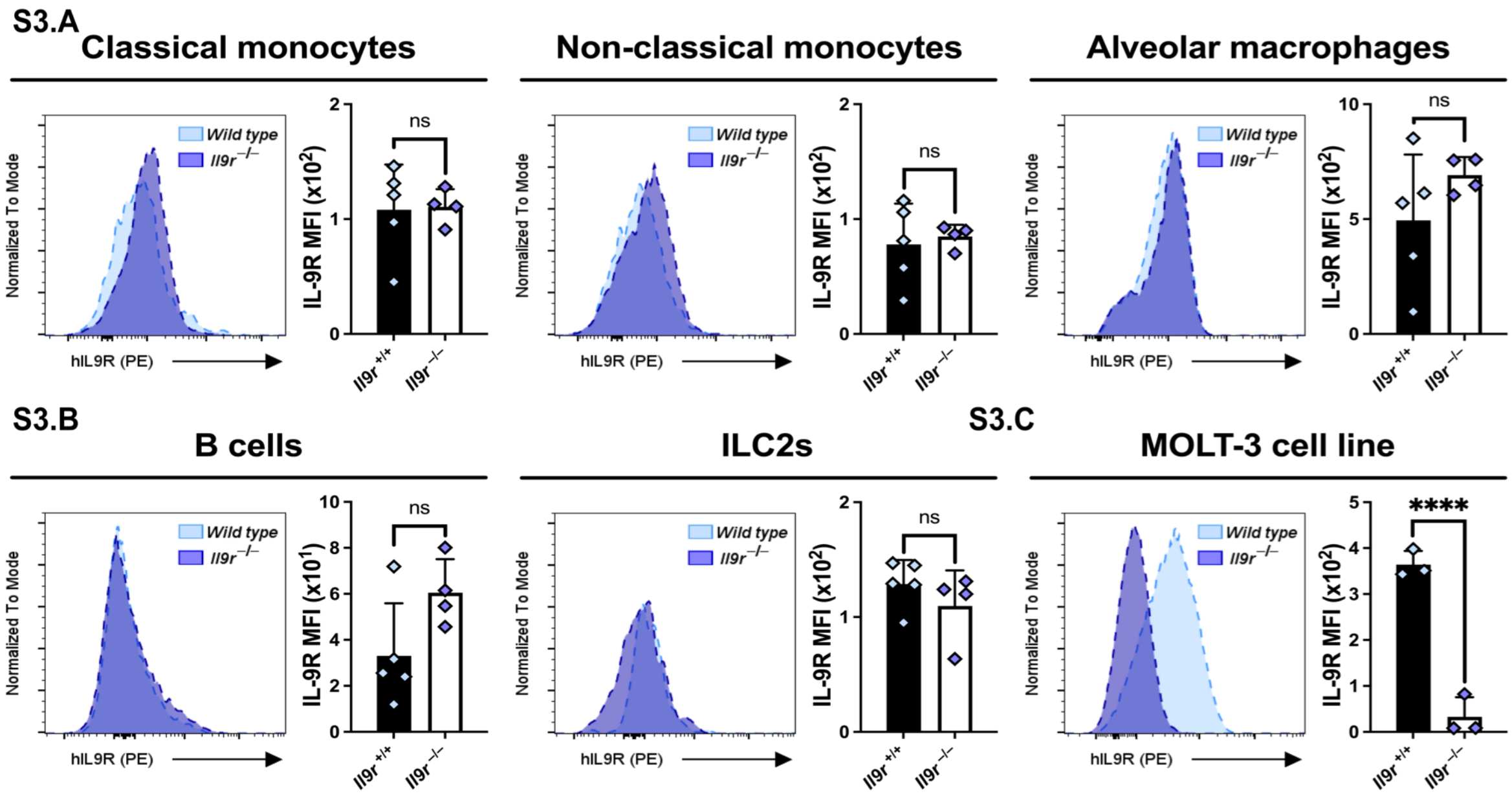

Supplementary Figure 3. A-C) Geometric mean fluorescence intensity (gMFI) of IL-9R on indicated immune cell types using an anti-human IL-9R IgG2b mAb (clone AH9R7) to stain (A-B) lung cells from naïve C57BL/6 wild-type and negative control *Il9r*$^{-/-}$ mice, and (C) Molt-3 immunized T cells with three technical replicates of wild-type and negative control *Il9r*$^{-/-}$ cells. (A-C) Histograms from representative mice (left) and columns showing the group averages with symbols indicating individual mice (right). Data are shown as mean ± s.d. Statistical analyses were performed using an unpaired *t*-test. Data are representative of two independent experiments.

Sup. Figure 4

S4.A

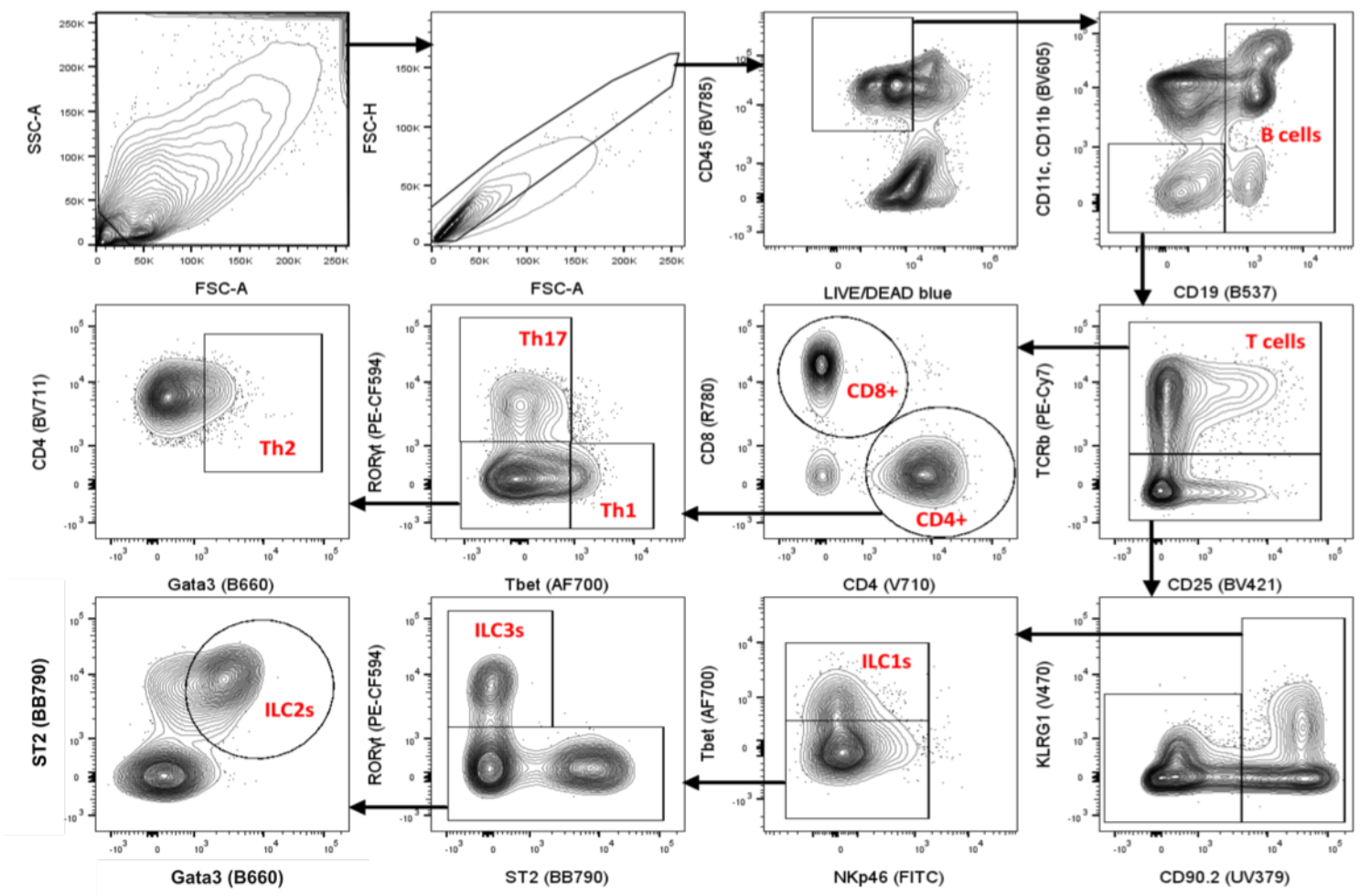

S4.B

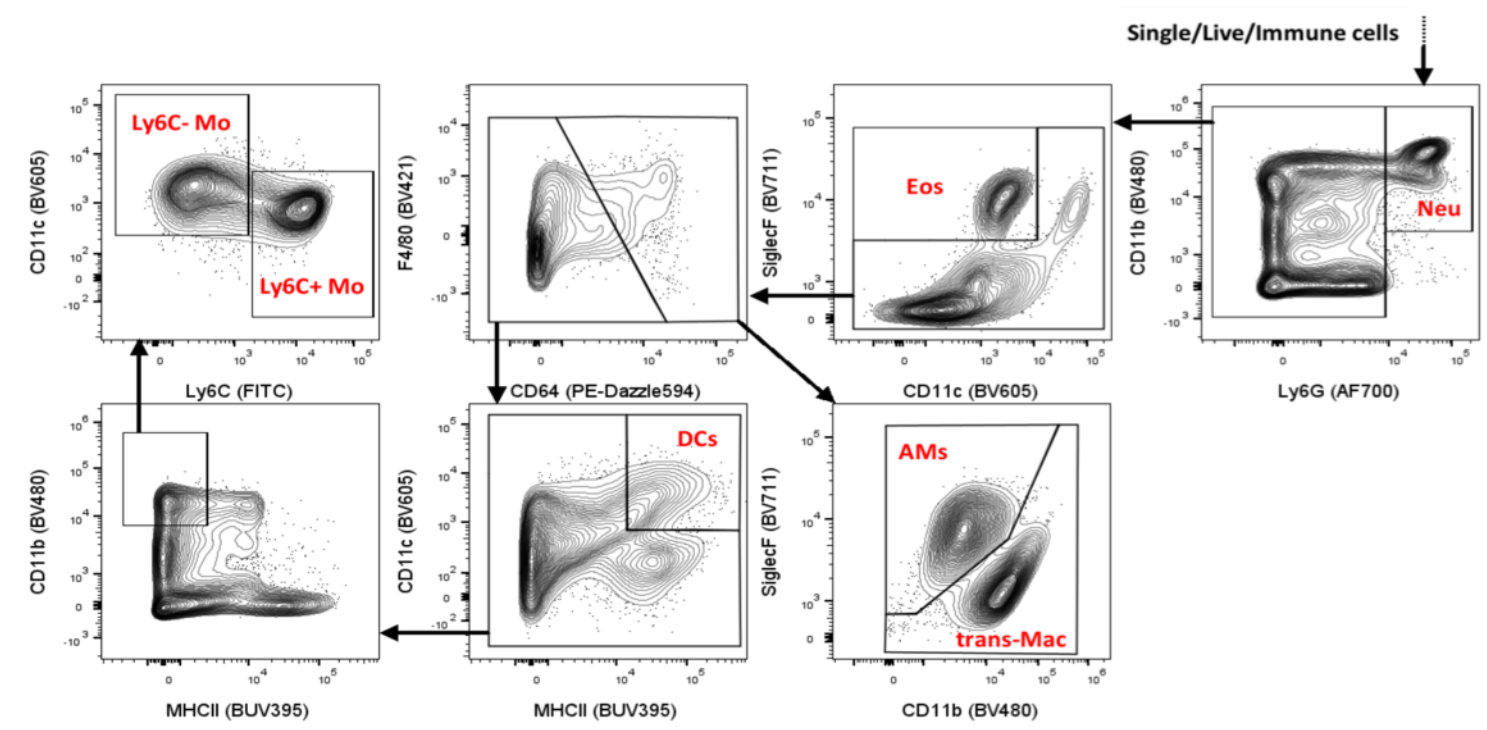

Supplementary Figure 4. A-B) Gating strategies for characterization of lymphoid (A) and myeloid (B) cells of the lung in mice after papain treatment.

Sup. Figure 5

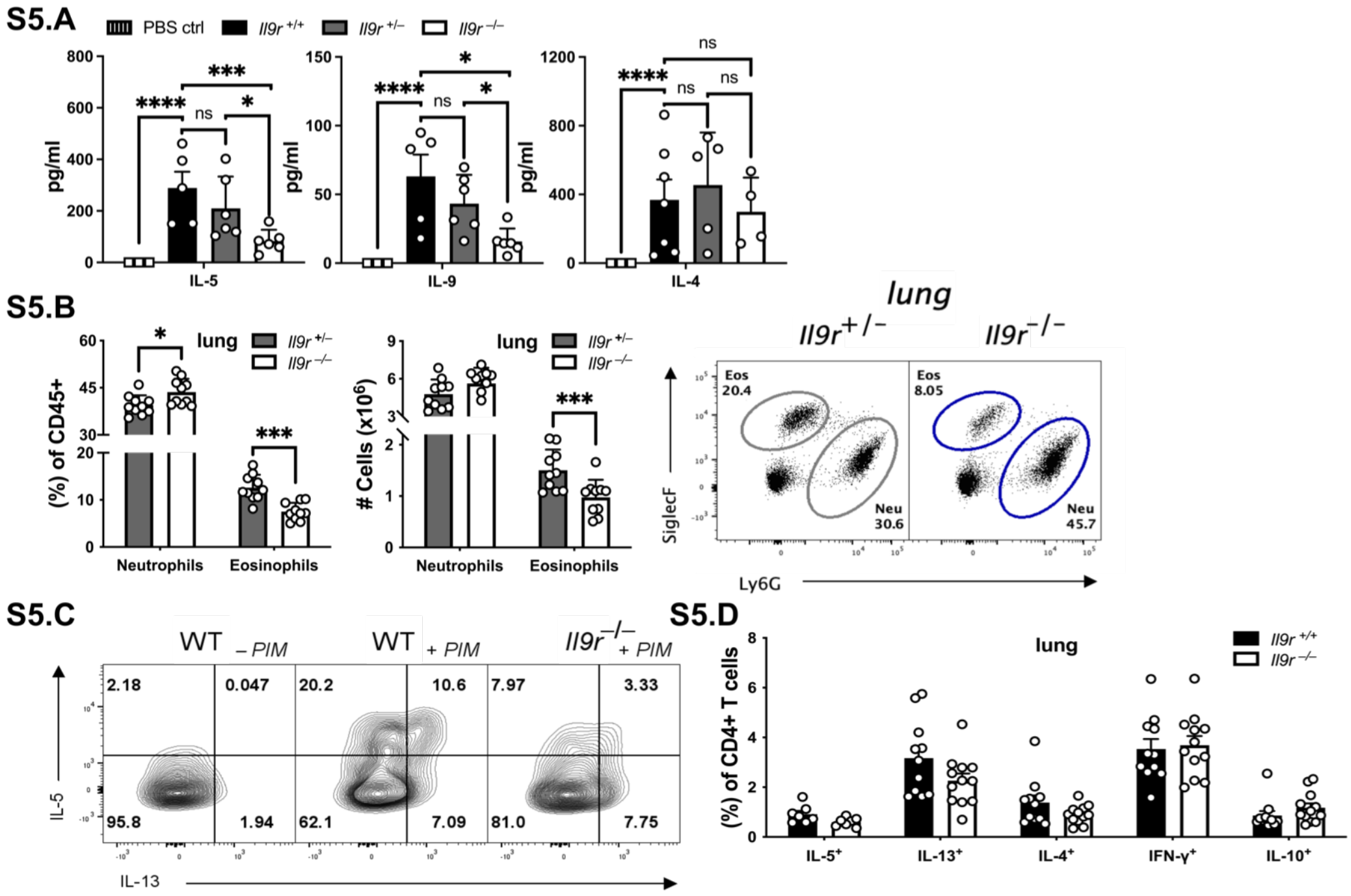

Supplementary Figure 5. A) Concentrations (pg/ml) of indicated cytokines in BAL. **B)** Frequency among CD45$^+$ lung cells and cell numbers of neutrophils and eosinophils and representative flow cytometry dot plots of *Il9r*$^{+/-}$ (grey) and *Il9r*$^{-/-}$ (white) mice. **C)** A representative flow cytometry dot plot of pre-gated lung ILC2s showing IL-5 and IL-13 expression without or after restimulation with PIM (PMA, Ionomycin and Monensin). **D)** Frequency of CD4$^+$ cytokine-producing T cells after ex vivo restimulation of wild-type (black) and stimulated *Il9r*$^{-/-}$ (white) mice. Data are shown either as mean ± s.d. when representative or as mean ± s.e.m. when pooled. Statistical analyses were performed using an unpaired *t*-test. Data are either representative of two independent experiments (A), three independent experiments (B-C) or pooled from two independent experiments (D).

Sup. Figure 6

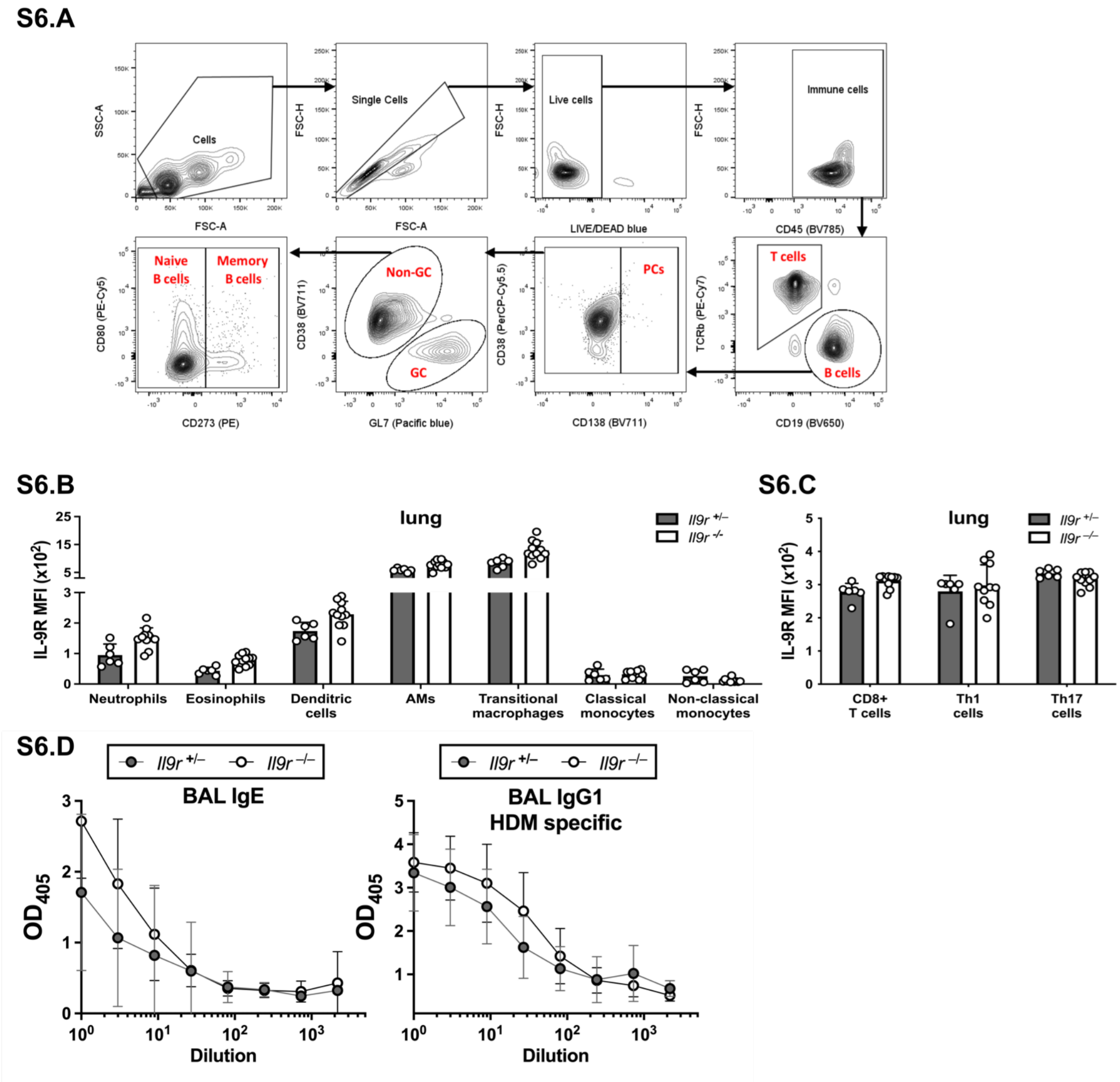

Supplementary Figure 6. A) Gating strategies for characterization of B cells in the lung of mice after HDM treatment. **B-C)** Geometric mean fluorescence intensity (gMFI) of IL-9R on indicated myeloid (B) and lymphoid (C) cells detected using the anti-mouse antibody in *Il9r*$^{+/-}$ and *Il9r*$^{-/-}$ mice as negative gating controls. **D)** Total BAL IgE and HDM-specific IgG1 levels. Data are shown as mean ± s.d. IC_{50} values were calculated for each individual mouse by nonlinear regression (four-parameter logistic fit) of ELISA dilution curves. Statistical analyses were performed using an unpaired *t*-test. Data are representative of two or three independent experiments.

Sup. Figure 7

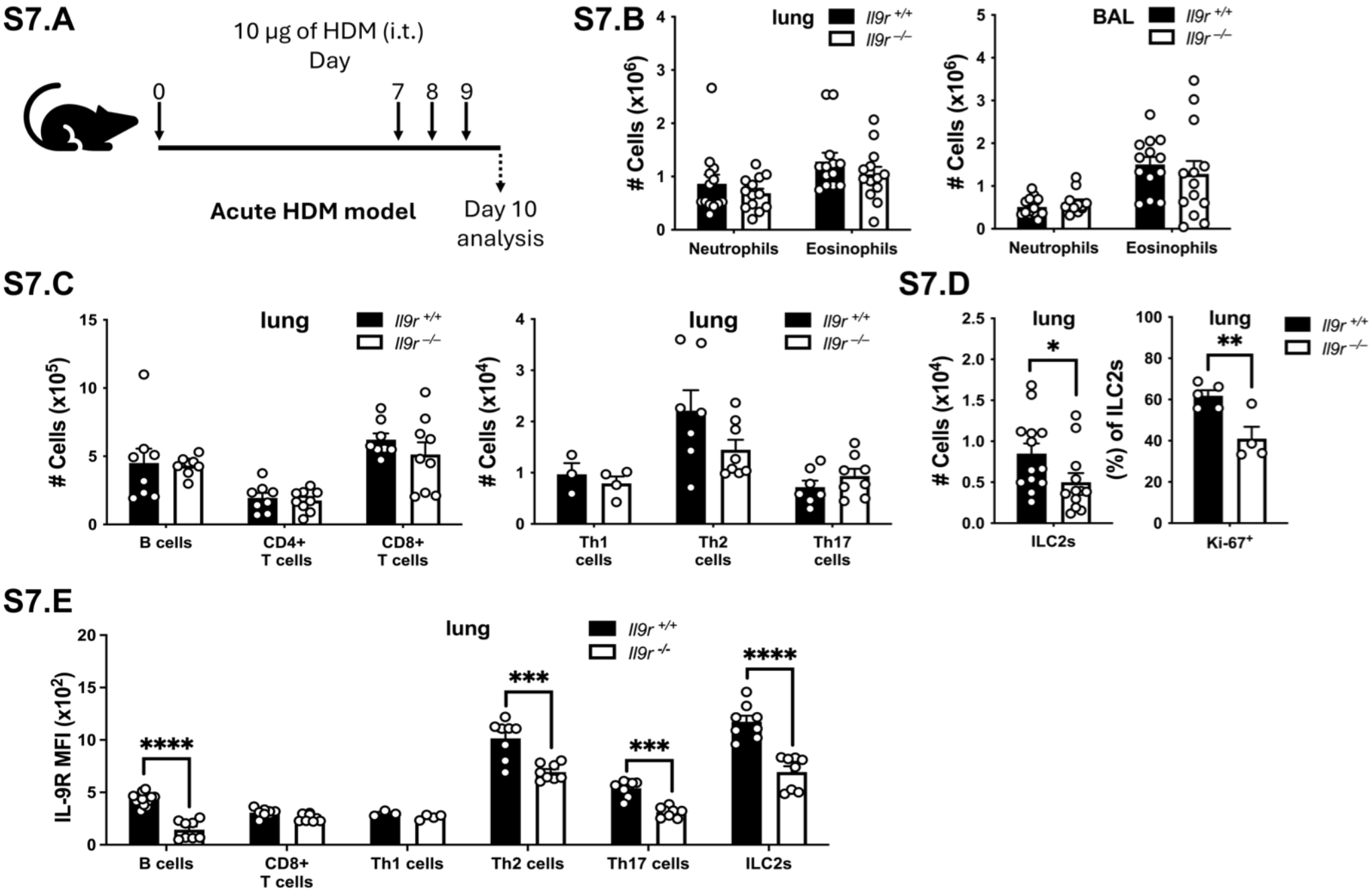

Supplementary Figure 7. A) Schematic representation of the acute HDM model. **B)** Quantification of neutrophils and eosinophils in the lung and BAL. **C)** Total cell numbers of adaptive lymphocytes and $CD4^+$ T-cell subsets (Th1:$CD4^+Tbet^+$, Th2: $CD4^+Gata3^+$, Th17:$CD4^+RORgt^+$) in the lung. **D)** Total cell numbers of ILC2s and percentage of Ki-67^+ ILC2s in the lung. **E)** Geometric mean fluorescence intensity (gMFI) of IL-9R on indicated cell types in the lung. Data are shown as mean ± s.e.m. Statistical analyses were performed using an unpaired *t*-test. Data are pooled from two or three independent experiments.

Sup. Figure 8

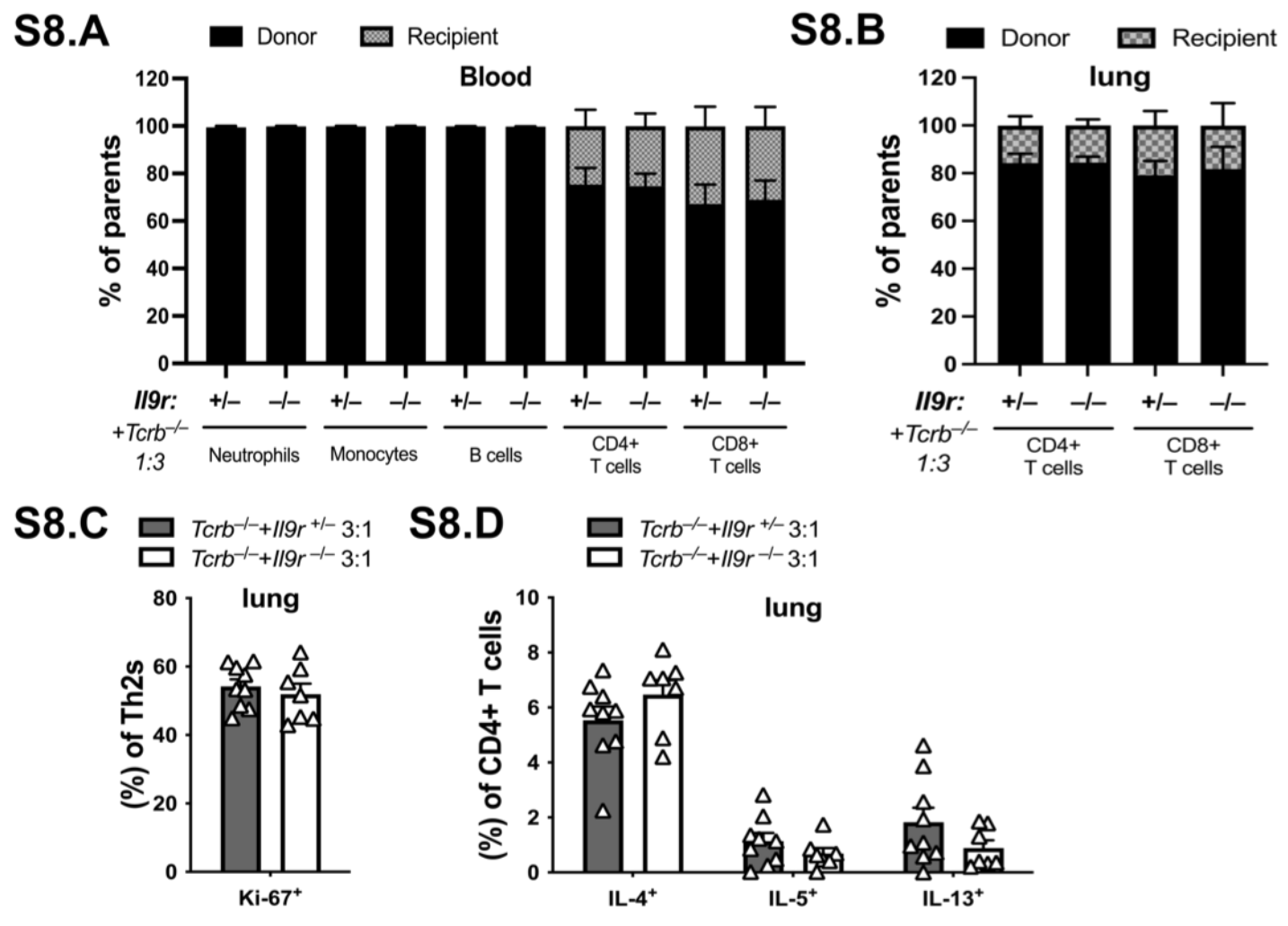

Supplementary Figure 8. A) Analysis of reconstitution of immune cells in the blood 8 weeks after bone marrow transfer. **B)** Analysis of reconstitution of T cells in the lung on the day of analysis. **C)** Percentage of Ki-67^{+} Th2s. **D)** Frequency of CD4^{+} cytokine-producing T cells after short ex vivo restimulation in challenged 3:1 *Tcrb*$^{-/-}$+*Il9r*$^{+/-}$ (grey) and 3:1 *Tcrb*$^{-/-}$+*Il9r*$^{-/-}$ (white) mice. Data are shown as mean ± s.e.m. Statistical analyses were performed using an unpaired *t*-test. Data are pooled from two independent experiments.
